# Physical activity, energy expenditure and sedentary parameters in overfeeding studies - a systematic review

**DOI:** 10.1186/s12889-018-5801-2

**Published:** 2018-07-21

**Authors:** Valerie Giroux, Soraya Saidj, Chantal Simon, Martine Laville, Berenice Segrestin, Marie-Eve Mathieu

**Affiliations:** 1 École de kinésiologie et des sciences de l’activité physique, Université de Montréal, P.O. Box 6128, Downtown Station, Montreal, Quebec H3C 3J7 Canada; 2 CARMEN, INSERM U1060/University of Lyon/INRA U1235, Lyon, France; 3 Human Nutrition Research Centre of Rhône-Alpes, Hospices Civils de Lyon, Lyon, France; 4 CHU Sainte-Justine, Montreal, Qc Canada

**Keywords:** Overfeeding, Physical activity, Exercise, Sedentariness, Energy expenditure, Assessment

## Abstract

**Background:**

It has been proposed that compensations in physical activity, energy expenditure and sedentary parameters can occur as a result of overfeeding studies in order to maintain body weight; however, the evidence has not yet been systematically reviewed.

**Methods:**

The current study systematically reviewed the literature on this subject to determine the common tools used in overfeeding studies and to explore whether overfeeding produces changes in physical activity, energy expenditure and sedentary parameters. Eight electronic databases were searched to identify experimental studies using keywords pertaining to overfeeding, exercise, physical activity and sedentariness. Articles included healthy adults (aged 18–64 years) participating in an overfeeding study that examined at least one parameter of sedentary, energy expenditure or physical activity. Of 123 full-text articles reviewed, 15 met the inclusion criteria.

**Results:**

The common tools used in overfeeding studies were doubly labeled water (*n* = 6), room calorimeter (*n* = 4), accelerometer (*n* = 7), pedometer (*n* = 3), radar sensor (*n* = 4) and survey (*n* = 1). Parameters partaining to energy expenditure increased between 7 to 50% with different overfeeding duration. Physical activity parameters, such as number of steps and spontaneous activity, increased or decreased significantly in three studies, while five studies showed no significant change. Sedentary parameters were examined by only one study and its results were not significant after 3 days of overfeeding. Methodological issues existed concerning the small number of studies, disparities in sedentary and physical activity parameters and various definitions of free-living experimental conditions and physical activity limits.

**Conclusions:**

There is actually a use of many tools and a large variation of parameters for physical activity in overfeeding studies. Contradictory findings showed changes in physical activity parameters following overfeeding and limited findings support the absence of changes in sedentariness. While energy expenditure parameters are more numerous and all show an increase after an overfeeding period, further studies are required to confirm changes in physical activity and sedentary parameters.

**Electronic supplementary material:**

The online version of this article (10.1186/s12889-018-5801-2) contains supplementary material, which is available to authorized users.

## Background

Obesity is rising at a epidemic rate and is a burden on the population, given that 70% of obese individuals struggle with numerous physiological disturbances such as metabolic complications, inflammation, dyslipidemia, hypertension [1] and increased mortality risk [2]. While a positive energetic balance is a crucial determinant of obesity development, some experimental studies simulated this stage by increasing energy intake beyond energy requirements to maintain body weight. These overfeeding studies aimed to unravel the physiological adaptation to nutrient excess and in particular the evolution of 1) changes in body composition; 2) possible alterations in carbohydrates, lipids or proteins metabolisms; 3) changes in endocrine functions; and 4) changes in energy metabolism and mitochondrial function [3–5].

In this context of experimental overfeeding, it is of major importance to focus simultaneously on energy intake and energy expenditure to observe their mutual influence. Neumann [6] and Gullick [7] were two researchers who performed early overfeeding studies on themselves. At that time, Neumann created the term ‘luxus consumption’ that is, the production of extra heat as a response to increased food intake. Today, overfeeding studies remained of interest in understanding the first adaptations resulting in weight gain. As recently reviewed, the average weight gain observed in most overfeeding studies are lower than expected, suggesting the presence of mechanisms that counteract the effects of excess energy intake [8]. In this field of investigation, small increases in resting metabolic rate and the thermic effect of food are mechanisms of interest, but only partially explain lower than expected body weight gain [9]. Consequently, some researches focused on adaptive thermogenesis, which includes resting energy expenditure and non-resting energy expenditure and explains the energy dissipation during overfeeding [10].

According to Schoeller [11], energy expended in physical activity (PA) is a component that accounts to a large degree of the variability in weight gain during overfeeding. Nevertheless, as Schutz [8] points out, explaining moderate weight gain only partially, the increase in physical activity thermogenesis cannot be the only mechanism at play. Levine et al.*,* [9] suggested in 1999 the existence of non-exercise activity thermogenesis (NEAT), defined as ‘thermogenesis’, that accompanies physical activities other than volitional exercise such as the activities of daily living, fidgeting, spontaneous muscle contraction, and maintaining posture when not recumbent. Measuring NEAT was a real challenge at the time. Nevertheless, technological advances put forward by many authors such as accelerometry now allow the measurement of some of these components in a daily lifestyle setting [12]. The aims of the current systematic review are thus 1) to examine the common tools measuring PA, energy expenditure and sedentary parameters in overfeeding studies and 2) to explore whether overfeeding produces changes in these parameters.

## Methods

The review was conducted in accordance with the Preferred Reporting Items for Systematic reviews and Meta-Analysis (PRISMA) statement guidelines [13]. Eligibility criteria are described in Table 1.

**Table 1:** PICOS (Participants, interventions, comparisons, outcomes, study design)

PICOS component	Details
Participants (P)	Adults aged 19–64 years with no eating disorders, no medication, non-smokers or light smokers and a body mass index ≥18 kg/m^2^
Interventions (I)	Overfeeding intervention (≥ 2 days) including at least one physical activity or sedentary parameter measurement
Comparisons (C)	Pre and post-overfeeding intervention
Outcomes (O)	Overfeeding, overeating, overnutrition, overnourishment, excessive eating, binge eating, physical activity, exercise, sports, sedentariness, physical inactivity
Study design (S)	Randomized control trials, non-randomized controlled trials and non-randomized non- controlled trials, prospective and observation

A search was conducted with no publication date or status restrictions. Studies were identified by searching 8 electronic databases: Medline (1966-present), Embase (1980-Present), CINAHL (1937-present), Scopus (1970-present), Web of Science (1980-present), CAB Abstracts (1973-present), PsycInfo (1806-present), and Cochrane controlled trials (1898-present). A filter was applied for publications in the English language only. The last search was conducted on July 12, 2017.

The following key words, presented in Table 2 and described in Table 3, and operators were used (search strategy: CAB Abstracts):

1. exp. overfeeding
2. exp. overeating
3. Overfeeding or over feeding, overeating or over eating, overnutrition or over nutrition, or overnourished or over nourished or excessive eating. (ab, ti)
4. 1 or 2 or 3
5. exp. physical activity
6. exp. sport
7. Physical activity, or exercise, or sport, or sedentary or sedentariness, or physical inactivity (ab, ti)
8. 5 or 6 or 7
9. 4 and 8

**Table 2:** Keywords included in the database search strategy

Eating type	Activity level
Overfeeding	Physical activity
Overeating	Exercise
Overnutrition	Sports
Overnourished	Sedentary
Excessive eating	Sedentariness
Binge eating	Physical inactivity
Overfeeding

**Table 3:** Definition of terms for overfeeding, physical activity and sedentariness

Term	Definition of variables
Overfeeding	Energy intake exceeding total energy expenditure over a given period of time
Physical activity	Any bodily movement produced by skeletal muscles that requires energy expenditure
Exercise	Regular and structured subsets of physical activity, performed deliberately and with a specific purpose such as preparation for athletic competition or improvement of some aspect of health
Sedentary behavior	Sedentary behaviors are behaviors characterised by a seated or reclining posture and a low energy expenditure ≤1.5 MET during waking hours
Physical inactivity	Activity level insufficient to meet present recommendations

Titles and abstracts of studies were independently screened by two authors (VG, SS) to determine a first selection of relevant papers. Studies had to meet the following criteria: 1) adult subjects only; 2) presence of an overfeeding protocol; and 3) physical activity, energy expenditure or sedentary level measurements. Disagreements between reviewers were resolved by consensus. Next, a detailed analysis of the papers by one reviewer (VG) led to their inclusion in this review. Studies with no data available relating to overfeeding were excluded. Studies with results only presented in a meeting abstract form were also excluded. Only studies reporting unrestricted physical activity were included, while studies reporting restricted physical activity were excluded (e.g., step count ≤4000 steps/day [14] or structured 30 min of bicycle per day). Studies were excluded on basis of eligibility criteria if there was a weakness in the use of PA tools (e.g., use of indirect calorimetry, but only for the resting metabolic rate) or a weakness in the overnutrition protocol (e.g., protocol duration for 1 day or ad libitum energy intake protocol).

Data collection was performed by one reviewer. There was no need to contact any authors for additional information.

Risk of bias was assessed using the Cochrane Collaboration’s tool for assessing risk of bias for sequence generation, allocation concealment, blinding outcome assessors, incomplete outcome data, selective outcome and other sources of bias [15]. Results are presented in an additional file (Additional file 1). Study selection inclusion was not influenced by the results of the risk of bias assessment. PA, energy expenditure and sedentary parameters were the outcomes of primary interest.

One author (VG) extracted the information into a spreadsheet that included the following: authors, date of publication, sample size, participant characteristics (age, sex, body mass index, body fat index, physical activity details, eating habits), setting, outcome measures (energy expenditure, physical activity parameters, sedentary parameters) and results. Results were converted into international units. Changes in energy expenditure were converted to percentages, if not initially provided.

## Results

**Fig. 1:**
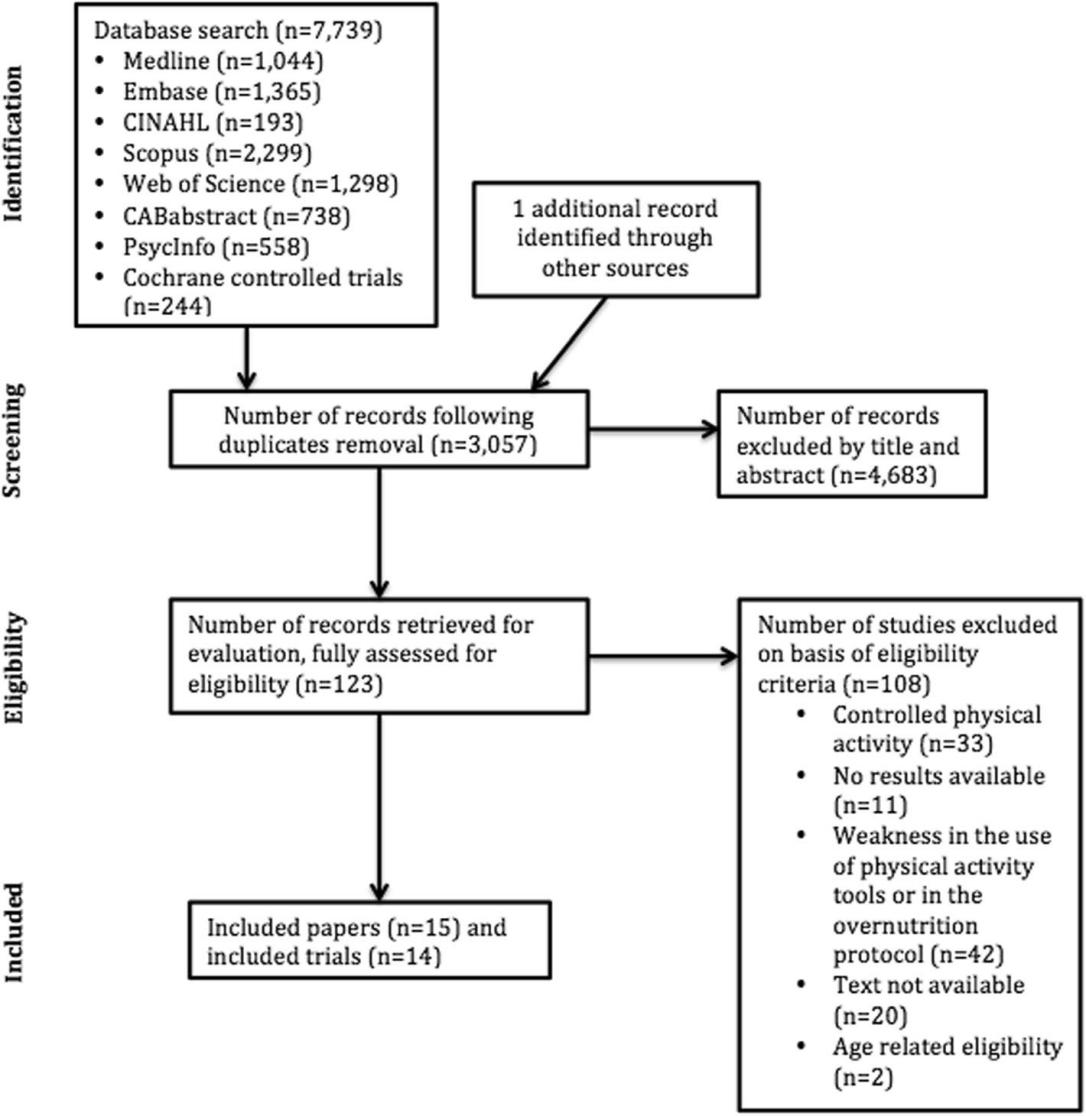
Systematic review flowchart

Figure 1 illustrates the systematic review flowchart following PRISMA guidelines [13]. The databases search yielded 7739 articles, 2934 of which were eliminated on the basis of their titles and abstracts alone. The full texts of 123 articles were subsequently retrieved, of which 15 articles gathering 14 trials met the inclusion criteria.

### Testing protocol

The 14 trials retrieved from this sytematic review took place between 1985 and 2015. The size of the studies varied between 4 [17] and 32 [24, 28] participants and all studies were within-person comparisons over time. Five overfeeding studies used a specific amount of calories for all subjects [9, 16–19]. Other studies opted for a personalized diet based on baseline energy requirements [20–29], calculated with estimations or direct measurements of resting energy expenditure, diet induced-thermogenesis and activity energy expenditure (see Table 4). There were variations in overfeeding duration ranging from 2 days [29] to 65 [20, 25] days. Regarding PA guidelines, 6 studies reported instructions of unrestricted PA [16–19, 24, 28], 5 studies asked their participants to avoid any specific PA [9, 20, 23, 27, 29], while the remaining 4 didn’t give any instructions concerning the practice of PA at all [21, 22, 25, 26]. Seven studies used accelerometers [16, 18, 20, 23–25, 27] six studies used doubly labeled water [9, 16, 17, 19, 20, 26] four studies used a room calorimeter [21, 22, 27, 29] and radar sensor [20, 23, 27, 29], three studies used a pedometer [24, 27, 28] and one study used a survey [25]. Finally, eight studies were in a free-living setting [9, 16, 19, 24–28] and seven were in a laboratory setting [17, 20–23, 27, 29].

**Table 4:** Studies assessing sedentary, energy expenditure or PA parameters with an overfeeding protocol

Reference (year)	Study design (N, RCT/nRCT)	Participants characteristics (Age range, sex, body mass index)	Overfeeding protocol	Setting	Physical activity indication	Tools and indicators	Results		
							Pre	Post	Change
Apolzan et al. (2014) [20]	25, RCT	- 18-35 yr. - 16 men, 9 women - 19.0-30.0 kg/m^2^	1.4× baseline energy requirement, for 65 days 3 groups: - low protein diet (5%) - normal protein diet (15%) - high-protein diet (25%) Based on 24-h of metabolic chamber direct measurments	Laboratory Participants resided on the inpatient unit for the entire study	Exercise prohibited	Doubly labeled water			
						• Total daily energy expenditure for 9 days (week 7–8) • Activity-related energy expenditure Calculated with sleeping metabolic rate and thermic effect of food (metabolic chamber)	n/a 417 ± 81	n/a 623 ± 159	• Not significant • **↑**50%
						Accelerometer			
						• Vector magnitude (counts) • Activity energy expenditure (kJ/day) RT3 accelerometer at the waist	102,60 ± 7,97 281 ± 29	132,60 ± 8,45 337.20 ± 26	• **↑**30% • **↑**20%
						Radar sensor			
						• Physical activity level	1.47 ± 0.06	1.56 ± 0.10	• **↑**6%
						• Spontaneous physical activity (kJ/day)	180 ± 21	n/a	• Not significant
						• Activity (% of active time/24 h) On day 1, 14 and 56 of the study	15.4 ± 0.9	n/a	• Not significant
Bray et al. (2015) [21]	25, RCT	−18-35 y - men and women −19.7-29.6 kg/m^2^	1.4× baseline energy requirement for 56 days 3 groups: - low protein diet (5%) - normal protein diet (15%) - high-protein diet (25%) Based on energy expenditure measured by doubly labeled water	Laboratory Participants resided in the Pennington Biomedical Research Center. Room calorimeter on week 8.	No indication	Room calorimeter			
						• Total daily energy expenditure	1993 ± 371 (kcal)	2137 ± 402	• **↑**8%
Dirlewanger et al. (2000) [22]	10, RCT	−20-26 y -women −19.3-25.3 kg/m^2^	1.4× of baseline energy requirement, for 6 days 1 group, 2 overfeeding diets: - hyperenergetic diet providing 40% excess energy as carbohydrates - hyperenergetic diet providing 40% excess energy as fat Based on resting energy expenditure measured by 45 to 60 min of indirect calorimetry × 1.3	Laboratory Room calorimeter on day 3	No indication	Room calorimeter			
						• Total daily energy expenditure	n/a	n/a	**↑** 7%
He et al. (2012) [23]	21, RCT	−42 y -men and women - 33.2 kg/m^2^	1.5× weight maintenance, diet for 3 days 1 group Specific to the inpatient unit and calculated based on body weight and sexe	Laboratory Participants were admitted to the Clinical Research Unit of the National Institute of Diabetes and Digestive and Kidney Diseases	Free walk allowed but exercise prohibited	Accelerometer			
						• Sedentary time (%) • Non-exercice activity (counts/min)	70.9 ± 12.9	72 ± 7.4	• Not significant
						Actical monitor worn at the waist, wrist and ankle	93.9 ± 21.5	68 ± 18.4	• Not significant
						Radar sensor			
						• Spontaneous physical activity (% of active time/24 h)	5.6 ± n/a	5.0 ± n/a	• Not significant
Joosen et al. (2005) [16]	25, nRCT	−19-36 y -women − 18.8 − 24.4 kg/m^2^	+ 50% more energy than the baseline energy requirement, for 14 days 1 group Calculated from basal metabolic rate measured with indirect calorimetry and PA level measured with accelerometry	Free living	Unrestricted	Accelerometer			
						• Physical activity indicator (Mcounts/day) Tracmor worn at the waist	6666 ± 1286	7177 ± 1645	• Not significant
						Doubly labeled water			
						• Total energy expenditure	10.18 ± 0.68	10.58 ± 1.00	• Not significant
Klein and Goran (1993) [17]	4, nRCT	-24-35 y -men −21.8-23.4 kg/m^2^	+ 6904 kJ/day, for 8 days 1 group Baseline indirect calorimetry measurements	Laboratory Participants were free to move around within the Clinical Research Center during the study with an access to a stationary bicycle ergometer.	Unrestricted	Doubly labeled water			
						• Total daily energy expenditure • Non-resting energy expenditure Calculated with resting metabolic rate (indirect calorimetry)	2384 ± 219 855 ± 190 (kcal/day)	2808 ± 291 1171 ± 262	• ↑18% • ↑42%
Levine et al. (2008) [18]	22, nRCT	−31-47 y - 12 women and 10 men - 19.0-38.0 kg/m^2^	+ 4184 kJ/day above weight maintenance, diet for 56 days 1 group Baseline period of 3 weeks during which the dietary intake provided was adjusted to maintain body weight gain	Free living	Unrestricted	Accelerometer			
						• Walking bouts (n/day) • Time engaged in walking (min/day) • Average distance of a walking bout (miles) • Free-living velocity (mph) PAMS system: 2 accelerometers (CXL02LF3-R, Crossbow technology) and 4 inclinometers (CXTA02, Crossbow Technology) on the trunk	47 ± 6 389 ± 106 0.18 ± 0.06 1.14 ± 0.20	47 ± 10 391 ± 116 0.15 ± 0.06 1.02 ± 0.20	• ↓1% • ↓1% • ↓ 21% • ↓ 12% Average of lean and obese group
Levine et al. (1999) [9]	16, nRCT	−25-36 y −12 men and 4 women	+ 4184 kJ/day, for 56 days 1 group Based on doubly labeled water measurements	Free living	Exercise prohibited	Doubly labeled water			
						• Total daily energy expenditure • Nonexercise activity-thermogenesis Calculated with basal metabolic rate and postprandial thermogenesis (indirect calorimetry)	2807 ± n/a 896 ± n/a	3361 ± n/a 1235 ± n/a (kcal)	• ↑12% • ↑38%
Muller et al. (2015) [24]	32, nRCT	−20-37 y -men −20.7-29.3 kg/m^2^	1.5× baseline energy requirement, for 7 days 1 group Based on a dietitian-guided dietary record, resting metabolic rate with indirect calorimetry and PA with the use of 24 h heart rate and accelerometry	Free living	Unrestricted	Accelerometer			
						• Activity energy expenditure (Kcal/d)	555 ± 328	580 ± 304	• Not significant
						Pedometer			
						• Steps/day	4785 ± 1417	4865 ± 1896	• Not significant
Pasquet et al. (1992) [25]	9, nRCT	−20-37 y -men −18.3-23.1 kg/m^2^	125 ± 46.6% of baseline habitual intakes, for 61–65 days 1 group Based on total energy expenditure measured with doubly labeled water	Free living	No indication	Survey			
						• Spontaneous activity	n/a	n/a	• ↓ 59%
						Accelerometer			
						• PA indicator At the wrist	4145 ± 1371	2440 ± 816	• ↓ 40% arm movement counts/24H
Siervo et al. (2008) [26]	6, nRCT	−32-58 y -men −18.8-24.1 kg/m^2^	1.6× baseline energy intake, for 21 days 1 group Baseline period of 3 weeks during which the dietary intake provided was adjusted to maintain body weight gain	Free living Participants were free to move around the Cambridge area	No indication	Doubly labeled water			
						• Total energy expenditure	11.1 ± 0.7	12.9 ± 0.8	**↑** 16%
Ravussin et al. (1985) [27]	5, nRCT	−22-27 y -men −19.0 − 23.9 kg/m^2^	1.6× baseline energy requirement, for 9 days 1 group Based on energy expenditure within a metabolic chamber plus an estimated 25% for PA:	Free living and laboratory Room calorimeter on day 1 and 9 of the study (5 m long × 2,5 m wide)	Exercise prohibited	Room calorimeter			
						• Daily energy expenditure (kJ/day)	9.751 ± 0.423	10.423 ± 0.584 11.789 ± 500	• **↑** 7% on the 2nd day • ↑ 21% on the 9th day
						Pedometer			
						• Steps/day	n/a	n/a	• Not significant
						Accelerometer			
						• Activity index	0.026 ± 0.003	0.031 ± 0.004	• Not significant
						Radar sensor			
						• Spontaneous PA (%)	0.026 ± 0.003	0.031 ± 0.004	• Not significant
Roberts et al. (1990) [19]	7, nRCT	-23-24 y -men −22.7 − 25.3 kg/m^2^	+ 4200 kJ/day, for 21 days One group Based on direct measurments of total energy expenditure components	Free living	Unrestricted	Doubly labeled water			
						• Total daily energy expenditure (kJ/day)	13,883 ± 774	14,665 ± 678	• Not significant
Schmidt et al. (2012) [28]	32, RCT	-25-35 y -men and women −16.9-25.5 kg/m^2^	1.4× baseline energy requirement, for 3 days 2 groups: - obesity prone individuals - obesity resitant individuals Based on calorimetry energy expenditure measurments	Free living	Unrestricted	Pedometer			
						• Steps/day	n/a	n/a	• ↓ (data not available) For both groups
Weyer et al. (2001) [29]	6, RCT	−21-33 y -men −19.5-25.5 kg/m^2^	200% baseline energy requirement, for 2 days 1 group, 2 diets: - ad libitum diet - overfeeding Based on a 24 h in a respiratory chamber after 3 weeks of weight maintenance diet	Laboratory Room calorimeter	Exercise prohibited	Room calorimeter			
						• Daily energy expenditure	n/a	n/a	• **↑** 9%
						Radar sensor			
						• PA energy expenditure (MJ/day)	n/a	n/a	• Not significant

N.B.: values presented are mean ± standard deviation. *PA* physical activity

### Room calorimeter

Concerning room calorimeter, Ravussin [27] showed a daily total energy expenditure (TEE) increase of 7% on the second day and 21% on the last day of a 9-day overfeeding protocol at 160% of maintenance requirements. Other studies have also reported increases in daily TEE of 9% at 2 days using 200% of energy requirement [29]; of 8% at 56 days using 140% individual baseline energy requirement [21]; and of 7% at 6 days using 130% of resting energy expenditure [22]. Measurements were made continuously for 22.5 h [27], 23 h [22] and 24 h [21, 29]. Participants were either not allowed to practice vigorous PA [27] and/or exercise [21], or had simply no indication in term of PA [22, 29]. Radar sensors were once added to the room calorimeter, but PA measurements were not used to estimate total energy expenditure [29].

### Doubly labeled water

Three studies using doubly labeled water revealed an increase in energy expenditure: 18% with an 8-day overfeeding protocol using + 6941 kJ/day [17]; 16% with a 21 days overfeeding protocol using 1.6× baseline energy requirement [26]; the other at 12% with a 56-day overfeeding protocol using + 4184 kJ/day [9]. Other studies showed no significant results with a 56-day overfeeding protocol using 140% of individual baseline energy requirement [20]; 21 days using + 4230 kJ/day [19] and 21 days at + 4230 kJ/day [16]. Activity-related energy expenditure has been reported by three studies with an increase of 38% [9], 42% [17] and 50% [20].

### Accelerometer

Using a short duration protocol (7 days overfeeding protocol at 150% energy needs), an increase of 30% in vector magnitude and 20% in activity energy expenditure (AEE) was measured by Apolzan [20]. In longer protocols, activity (arm movement count/24H) showed a 40% decrease at 61–65 days at 125% of baseline habitual intake [25]. In addition, Levine [18] showed decreases of 1% in walking bouts and time engaged in walking, of 12% in free living velocity, and of 21% in average distance of a walking bout (miles) after 56 days of 4184 kJ/day above weight maintenance feeding. Other results have not been significant: activity AEE at 7 days with 150% of energy needs [24]; sedentary time and non-exercise activity at 3 days at 150% of weight maintenance diet [23]; PA after 14 days at + 4230 kcal/day [16]; and activity index at 9 days with 160% of maintenance requirements [27].

### Pedometer

In a protocol of 3 days at 140% of estimated basal energy needs [28], overfeeding was associated with a significant decrease in the number of steps of participants with BMIs between 16.9 and 25.5 kg/m^2^ (data not presented). Other studies have shown no difference in step counts at 7 days at 150% of energy needs [24] and at 9 days at 160% of maintenance requirements [27].

### Radar sensor

Radar motion detectors that continuously monitored the subject’s movement in the room calorimeter detected an increase in PA levels of 6% at 56 days at 140% individual baseline energy requirement [20]. These radar sensor measurements had no significant results on other PA parameters: activity [20]; spontaneous PA [20, 23, 27]; and physical activity energy expenditure [29].

### Survey

The survey, consisting of a time-allocation survey done by local assistants who recorded activities, postures and pace minute by minute for 24 h, revealed a decrease of 59% in spontaneous activity using either 61-or 65-day overfeeding protocols at 125% energy requirement [25].

## Discussion

The first aim of this review was to investigate the common tools measuring PA, energy expenditure and sedentary parameters in overfeeding studies. Ultimately, 15 papers assessed PA, energy expenditure or sedentariness with 1 tool (*n* = 8) or a combination of 2 to 4 different tools (*n* = 7) using room calorimeter, doubly labeled water, accelerometer, pedometer, radar sensor and survey. The 20 parameters identified are diversified, which provided a wide range of results that can be interpreted following overfeeding but also challenging their comparison. The second aim of this review was to explore whether overfeeding modulate these parameters: PA parameters were maintained, increased and decreased and energy expenditure parameters were increased or maintained. Only one study assessed sedentary parameters and its result indicated a maintenance level.

This systematic review first aimed to determine any preferential use of tools or specific parameters in overfeeding studies. The pedometer was the first tool to be used in an overfeeding study in 1967 [30]. Then appeared the metabolic chamber in 1971 [31]. Decades later, Ravussin et al. [27] introduced the accelerometer and the radar sensor and at the same time, a combination of tools for a single study. Finally, the last tool that emerged was the doubly labeled water in 1990 [19]. In this systematic review, these findings show that the accelerometer is the most common tool (*n* = 7), followed by doubly labeled water (*n* = 6). However, room calorimeter (*n* = 4), radar sensor (n = 4), pedometer (*n* = 3) and survey (*n* = 1) are less common. A combination of tools was used in 7 studies without any similarities among them.

In terms of parameters, this review identified 20 of them and what emerged was that there was no consensus on preferential parameters. Some tools allowed only one parameter, such as TEE (MJ/day) for the room calorimeter, as well as number of steps for the pedometer. The most popular parameter of doubly labeled water is daily energy expenditure [9, 16, 17, 19, 20, 26], while activity-related energy expenditure or its equivalent is also reported three times [9, 17, 20]. There was only one study that presented parameters of both physical activity and sedentary parameters [23], despite the fact that sedentariness and physical inactivity are two distinct concepts and that both contribute independently to excess body weight gain [32]. In a study conducted by Knudsen [33], inactivity and overfeeding have led to insulin sensitivity impairment. Furthermore, a high amount of sedentariness had an impact on morbidity regardless of PA level [34–36]. However, most of the negative effects of a short-term overfeeding combined with a daily-reduced number of steps are counteracted by physical exercise [14]. Therefore, by not considering PA, exercise, sedentariness and physical inactivity at the same time, there was a lack in the complete interpretation of what was happening in a positive energy balance in adult studies.

The second aim of this review was to explore changes in PA, energy expenditure and sedentary parameters following an overfeeding period. Our results indicated that there might be some changes in PA and energy expenditure parameters while sedentary parameters appeared to be maintained. For PA and energy expenditure parameters, changes were not significant (*n* = 13), increasing (*n* = 15) or decreasing (*n* = 7) with overfeeding, considering that each study could have more than one parameter. The study that appears to have had the greatest impact on PA parameters is that of Alpozan et al. [20] with an increase of 50% in activity-related energy expenditure and Pasquet et al.*,* [25] which showed decreases of about 60% in spontaneous physical activity and 40% in physical activity indicator (counts/day). Other decreases in PA parameters were related to walking characteristics and varied between 1 and 21% [18]. The decreases involved a greater change in the distance traveled (− 21%) compared to the daily walking time (− 1%), suggesting a less efficient walk and a modulation of energy efficiency. This joins the theory that overfeeding produces change in NEAT [9]. These results also show the importance of evaluating changes in a free living setting.

Increases energy expenditure parameters ranged from 6 to 60% and most were related to the measurement of daily energy expenditure. This wide difference in energy expenditure results could be explained by guidelines given to participants about their PA practice and the space in which the participant could move (Table 4). Interestingly, these increases were observed both in a free living context [9, 20, 26, 27] and when the study was conducted in an inpatient unit [17, 21, 22, 27, 29]. For sedentary behaviors, only one study used this parameter and its changes were not significant [23] just like PA in this study. Other researchers highlight that the duration of protocol and amount of overfeeding are two major factors that produced changes in these parameters [37, 38]. A review conducted by Westerterp [39] using doubly-labelled water found that there was no effect on PA level when overfeeding was lower than twice the maintenance requirements, a finding however in contradiction with some of the study used in the current review [20, 26].

**Fig. 2:**
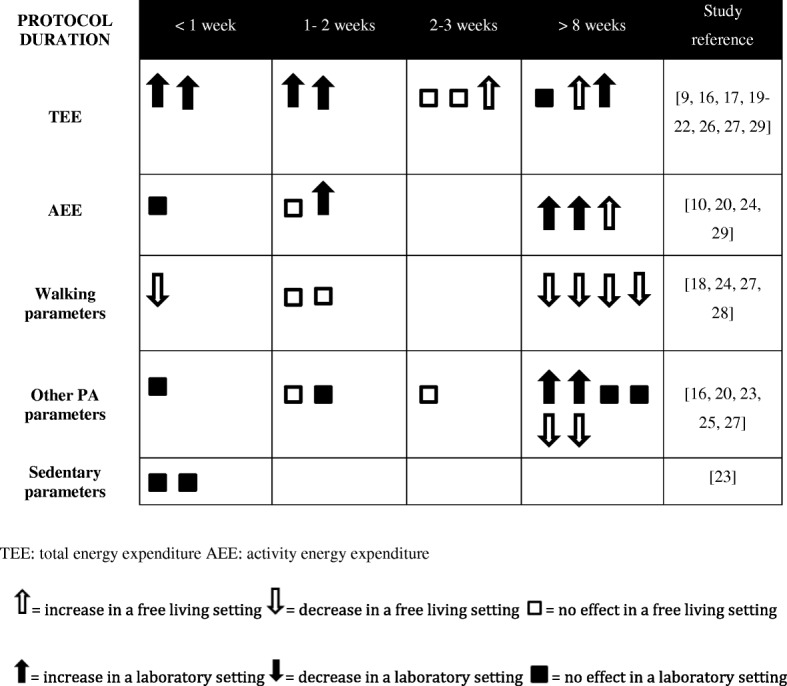
Effects of protocol duration on energy expenditure, physical activity and sedentary parameters in a free living or a laboratory setting

Interesting elements emerge when results were compared according to duration of the overfeeding protocol (Fig. 2). Durations of overfeeding varied greatly between different studies. As mentioned by Joosen and Westerterp [37], the overfeeding period should be long enough to expect an increase in excess body weight. We suggest that it can be the same with changes in PA and sedentary parameters. In fact, it seems that there were more changes when protocol duration exceeded 8 weeks, even if this impact can also be due to a greater number of free living settings with long overfeeding protocols.In fact, short-duration studies (< 1 week) are mostly done in laboratory. Figure 2 illustrates that TEE was either non-significant or increased with overfeeding. This increase in TEE seemed not to be caused solely by an increase in AEE but was rather multifactorial and possibly linked to the NEAT theory described previously. In fact, three studies performed assessment of both TEE and PA parameters [20, 27, 29]: they all indicated an increase in TEE while PA was maintained or increased (6 to 30%). Walking parameters were maintained and decreased with overfeeding, while other PA parameters were more divergent. For this parameter, a BMI ≥ 30 kg/m^2^ could lead to less pronounced changes, as it was found in the Schmidt et al. [28] overfeeding study comparing obesity prone and obesity resistant individuals. Interestingly, Levine and his colleagues carried out the same protocol of overfeeding (time, duration and setting) in 1999 and 2008, except that in the first study, the exercise was prohibited and in the second, physical activity was unrestricted [9, 18]. Again, a mechanism for dissipating excess energy consumption seems to exist, since there was an increase in TEE in the first one and a decrease in walking parameters in the second. These latter results agree with the NEAT theory, but measurements with an accelerometer was therefore incomplete as the non-volitional part could only be verified by radar sensors for a better understanding of its mechanisms.

According to Fig. 2, contradictions occur between increase in TEE and the other parameters. In the case of Apolzan et al. [20], there was no modification in total daily energy expenditure, but an increase in vector magnitude, AEE and physical activity while exercise was prohibited. Pasquet et al. [25], on the contrary, observed an increase in daily energy expenditure, but no significant results for any PA results assessed by pedometer, accelerometer and radar sensors. TEE’s ability to influence weight gain depends on the increase or decrease in PA and sedentary parameters and hence supports the importance of having precise and complementary parameters. Therefore, this table points out the need to further explore sedentary parameters in long-term overfeeding studies. This is even more important since there was a favorable association between breaks in sedentary time, triglycerides [40] and waist circumference [41], two biomarkers clustered into a metabolic risk.

Studies included in this review did not all agree on the same aims. Some focussed on energy expenditure [16, 19, 21, 22, 25–27], or on PA [18, 20, 23, 28] and among these, one study also aimed to measure sedentariness results [23]. Other studies had other aims such as to identify changes in metabolism [17, 29], thermogenic responses [9, 25], basal metabolic rate [29] and leptin levels [22]. These differences among studies could have an impact on the outcomes, which need to be examined.

The different purposes of each paper can explain the variability in the choice of tools and parameters. Kelly et al.*,* [42] pointed out that there is no ‘gold standard’ in terms of tool choice for PA and sedentary measurements: it all depends on the PA or sedentariness aspect of interest. While the goal of each study differed, it was unexpected to see a consensus regarding the use of a single tool. Nevertheless, questionnaires have limited reliability and validity when they are not for 1) indicating conditions where an increase in PA would be beneficial and 2) monitoring changes in population activity [43]. In fact, self-reporting of PA were overestimated compared to direct measurement tools such as doubly labeled water and accelerometry [44]. This potentially explains why only one study used a survey. If we consider overfeeding studies for the purpose of preventing obesity, then the room calorimeter, which allows the assessment of different components of TEE and thereby, energy substrate utilization, appears to be the tool of choice, as emphasized by Lam and Ravussin [45]. Conversely, if we want to understand why predictive models of weight loss or gain are inaccurate, then measurements of these parameters under free-living conditions could be more accurate. To do so, doubly labeled water and accelerometry are two devices of choice. Furthermore, interest on the combination of tools seems to be emerging [46]. Seven studies in this review used more than one tool for the overfeeding part of the protocol. However, these additions did not necessarily result in improvements in estimating EE [47] but did improve the accuracy of meaningful PA outcomes such as METs/hour and time spent in moderate to vigorous PA [46]. This combination is now important to consider since Pontzer et al.*,* [48] found that compensatory mechanisms that modulate energy expenditure occur with high intensity. In fact, above moderate activity levels, total energy expenditure plateaued.

The current review highlights a wide diversity in participant characteristics subjected to overfeeding protocols. It is now commonly known that confounding factors for energy expenditure include both participant aspects such as age [49], gender [50], genetic [51] and dietary nutriments such as food composition [38, 52, 53] and the type of fat in the diet [54]. With a homogeneous age group of adults, this review did not allow us to observe a difference for this confounding factor. Regarding gender, some articles in this review included only men [17, 19, 24–26, 29], others only women [16, 22], and some of them both [9, 18, 20, 21, 23, 28]. There is insufficient information for interpretation when controlling for energy expenditure and gender in this review, considering that there was only one study examining females and energy expenditure parameters. Finally, unconventional dietary nutriments were performed by Bray et al. [21] with a protein overfeeding and Dirlewanger et al. [22] with a carbohydrate and a fat overfeeding. With the increase of food intake, TEE increase through the processing of ingested food [55]. Protein overfeeding may increase even more TEE by increasing postprandial thermogenesis, which implies a significant bias on the results [55]. A brief period of high fat overfeeding impairs glycemic control [56] as well as carbohydrate overfeeding [53]. These changes in glucose blood concentration interfere with hormonal regulation and lead to fat production and accumulation [53]. Furthermore, some hormones increase TEE, such as leptin [57] which is increased following overfeeding [58].

### Limitations

The main limitation in this systematic review concerns the unreported or estimated PA measurements, potentially leading to overestimation of PA measurements. In addition, this makes the comparisons between studies more complex. Furthermore, the PA guidelines the volunteers had during the overfeeding studies and that can varied from one study to the other might have influenced energy expenditure results. Food intake was assessed in both laboratory conditions and free-living conditions. The first is known to be a rigorous method of assessing energy intake and the other may lead to underreporting and biased results [59]. According to Westerterp [38], the ideal measure of energy expenditure in overfeeding studies may be in non-restrictive conditions and with an increase in caloric ingestion for at least 1 week. In this case, 60% of studies included in this review were performed in free-living conditions and 66% respected a duration of more than 1 week. There is clearly a lot more work to be done to elucidate the effects of overfeeding on PA, energy expenditure and sedentariness, starting with direct measurments of these three components in a single study. In fact, no study to date has investigated them altogether.

## Conclusions

The investigation of PA, energy expenditure and sedentary measurements in an overfeeding context shows the use of various tools as well as a technological advance putting forward their uses in laboratory but also in free-living context. This systematic review draw a good state of the literature, as it is performed in eight databases and followed the PRISMA guidelines. Adaptations, both increase and decrease, seemed to occur in PA parameters following overfeeding. This might have been influenced by duration of the overfeeding protocols. An interesting consideration may be an “overfeeding % x duration protocol” factor for a global view of the overfeeding protocol. Unfortunately, 5 studies included in this review gave a specific amount of energy intake in excess instead of an overfeeding percentage, thus making it impossible to compare its effects on energy expenditure, PA and sedentary parameters. As there are a relatively small number of heterogeneous studies included in this review (*N* = 15), these results should be interpreted with caution. Thus, the development of the full potential of certain tools such as the accelerometer must be achieved. There was only one study that specifically assessed sedentariness. A logical next step for future trials would thus be to include sedentary parameters more frequently.

## Additional file

Additional file 1 *Risk of Bias Assessment.* Risk of bias assessed for sequence generation, allocation concealment, blinding outcome assessors, incomplete outcome data, selective outcome and other sources of bias. (DOC× 133 kb)

## Abbreviations

AEE: Activity energy expenditure
BMI: Body mass index
NEAT: Non-exercise activity thermogenesis
PA: Physical activity
TEE: Total energy expenditure
