# Supplementary material for: Physical activity, energy expenditure and sedentary parameters in overfeeding studies - a systematic review
Source: BMC Public Health. 2018 Jul 21;18:903. doi: 10.1186/s12889-018-5801-2 (PMC6054727; doi:10.1186/s12889-018-5801-2)
Supplement: Supplementary file 1 — Risk of Bias Assessment. Risk of bias assessed for sequence generation, allocation concealment, blinding outcome assessors, incomplete outcome data, selective outcome and other sources of bias. (DOC× 133 kb) [file 12889_2018_5801_MOESM1_ESM.docx]

RISK OF BIAS ASSESSMENT

|  | **Sequence generation** | **Allocation concealment** | **Blinding participants and personnel** | **Blinding outcome assessors** | **Incomplete outcome data** | **Selective outcome** | **Other source of bias** |
| --- | --- | --- | --- | --- | --- | --- | --- |
| Apolzan et al. (2014) | High risk | Unclear risk | High risk | High risk | High risk | Low risk | High risk |
| Bray et al. (2015) | Unclear risk | Unclear risk | High risk | High risk | High risk | Low risk | Low risk |
| Dirlewanger et al. (2000) | Unclear risk | Unclear risk | High risk | High risk | Low risk | Low risk | Unclear risk |
| He et al. (2012) | Unclear risk | Unclear risk | High risk | High risk | Low risk | Low risk | Low risk |
| Joosen et al. (2005) | Unclear risk | Unclear risk | High risk | High risk | Low risk | Low risk | Unclear risk |
| Klein and Goran (1993) | Unclear risk | Unclear risk | High risk | High risk | Low risk | Low risk | High risk |
| Levine et al. (2008) | Unclear risk | Unclear risk | High risk | High risk | Low risk | Low risk | High risk |
| Levine et al. (1999) | Unclear risk | Unclear risk | High risk | High risk | Low risk | Low risk | Low risk |
| Muller et al. (2015) | Unclear risk | Unclear risk | High risk | High risk | Low risk | Low risk | Low risk |
| Pasquet et al. (1992) | High risk | Unclear risk | High risk | High risk | High risk | Low risk | Unclear risk |
| Siervo et al. (2008) | Unclear risk | Unclear risk | High risk | High risk | Low risk | Low risk | Low risk |
| Ravussin et al. (1985) | Unclear risk | Unclear risk | High risk | High risk | Low risk | High risk | Low risk |
| Roberts et al. (1990) | Unclear risk | Unclear risk | High risk | High risk | Low risk | Low risk | High risk |
| Schmidt et al. (2012) | Unclear risk | Unclear risk | High risk | High risk | Unclear risk | High risk | Low risk |
| Weyer et al. (2001) | Unclear risk | High risk | High risk | High risk | Low risk | Low risk | Low risk |

| **Study : Apolzan et al. (2014)** | | |
| --- | --- | --- |
| **Entry** | **Authors’ judgement** | **Support of judgement** |
| *Selection bias* | | |
| **Random sequence generation** | High risk. | Quote : «participants were enrolled from June 2005 through October 2007 on a rolling basis and not in cohorts. » |
| **Allocation concealment** | Unclear risk. | No information |
| *Performance bias* | | |
| **Blinding of participants and personnel** | High risk. | Open label |
| *Detection bias* | | |
| **Blinding of outcome assessment** | High risk. | Open label |
| *Attrition bias* | | |
| **Incomplete outcome data addressed** | High risk. | Numbers randomized into each intervention group are not clearly reported. |
| *Reporting bias* | | |
| **Selective reporting** | Low risk. | All prespecified outcomes were reported. |
| *Other bias* | | |
| **Other source of bias** | High risk. | Exercise is prohibited. Physicial activity related to lifestyle is not considered. |

| **Study : Bray et al. (2015)** | | |
| --- | --- | --- |
| **Entry** | **Authors’ judgement** | **Support of judgement** |
| *Selection bias* | | |
| **Random sequence generation** | Unclear risk. | Quote : «participants were enrolled from June 2005 through October 2007» |
| **Allocation concealment** | Unclear risk. | No information |
| *Performance bias* | | |
| **Blinding of participants and personnel** | High risk. | Open label |
| *Detection bias* | | |
| **Blinding of outcome assessment** | High risk. | Open label |
| *Attrition bias* | | |
| **Incomplete outcome data addressed** | High risk. | Quote : « Participants were randomly assigned to one of 3 different protein diet ». Numbers randomized into each intervention group are not clearly reported |
| *Reporting bias* | | |
| **Selective reporting** | Low risk. |  |
| *Other bias* | | |
| **Other source of bias** | Low risk. | Exercise is prohibited. |

| **Study : Schmidt et al. (2012)** | | |
| --- | --- | --- |
| **Entry** | **Authors’ judgement** | **Support of judgement** |
| *Selection bias* | | |
| **Random sequence generation** | Unclear risk. | No information |
| **Allocation concealment** | Unclear risk. | No information |
| *Performance bias* | | |
| **Blinding of participants and personnel** | High risk. | Open label |
| *Detection bias* | | |
| **Blinding of outcome assessment** | High risk. | Open label |
| *Attrition bias* | | |
| **Incomplete outcome data addressed** | Unclear risk. | Numbers randomized into each intervention group are not clearly reported |
| *Reporting bias* | | |
| **Selective reporting** | High risk. | Not all prespecified outcomes were reported |
| *Other bias* | | |
| **Other source of bias** | Low risk. | None |

| **Study : Dirlewanger et al. (2000)** | | |
| --- | --- | --- |
| **Entry** | **Authors’ judgement** | **Support of judgement** |
| *Selection bias* | | |
| **Random sequence generation** | Unclear risk. | No information |
| **Allocation concealment** | Unclear risk. | No information |
| *Performance bias* | | |
| **Blinding of participants and personnel** | High risk. | Open label |
| *Detection bias* | | |
| **Blinding of outcome assessment** | High risk. | Open label |
| *Attrition bias* | | |
| **Incomplete outcome data addressed** | Low risk. | There is only one intervention including all participants. |
| *Reporting bias* | | |
| **Selective reporting** | Low risk. | All prespecified outcomes were reported |
| *Other bias* | | |
| **Other source of bias** | Unclear risk. | No information about physical activity indication. |

| **Study : He et al. (2012)** | | |
| --- | --- | --- |
| **Entry** | **Authors’ judgement** | **Support of judgement** |
| *Selection bias* | | |
| **Random sequence generation** | Unclear risk. | Quote : « Thirty-one volunteers were screened for this study form October 2007 to July 2009. Eight subjects were excluded for not meeting the criteria and other reasons » |
| **Allocation concealment** | Unclear risk. | No information |
| *Performance bias* | | |
| **Blinding of participants and personnel** | High risk. | Open label |
| *Detection bias* | | |
| **Blinding of outcome assessment** | High risk. | Open label |
| *Attrition bias* | | |
| **Incomplete outcome data addressed** | Low risk. | There is only one intervention including all participants. |
| *Reporting bias* | | |
| **Selective reporting** | Low risk | All prespecified outcomes were reported |
| *Other bias* | | |
| **Other source of bias** | Low risk. | Subjects were instructed not to exercice |

| **Study : Joosen et al. (2005)** | | |
| --- | --- | --- |
| **Entry** | **Authors’ judgement** | **Support of judgement** |
| *Selection bias* | | |
| **Random sequence generation** | Unclear risk. | No information |
| **Allocation concealment** | Unclear risk. | No information |
| *Performance bias* | | |
| **Blinding of participants and personnel** | High risk. | Open label |
| *Detection bias* | | |
| **Blinding of outcome assessment** | High risk. | Open label |
| *Attrition bias* | | |
| **Incomplete outcome data addressed** | Low risk. | There is only one intervention including all participants. |
| *Reporting bias* | | |
| **Selective reporting** | Low risk. | All prespecified outcomes were reported |
| *Other bias* | | |
| **Other source of bias** | Unclear risk. | Quote : « subjects maintained their normal lifestyles (work, education, sports participation) throughout the study » |

| **Study : Klein and Goran (1993)** | | |
| --- | --- | --- |
| **Entry** | **Authors’ judgement** | **Support of judgement** |
| *Selection bias* | | |
| **Random sequence generation** | Unclear risk. | No information |
| **Allocation concealment** | Unclear risk. | No information |
| *Performance bias* | | |
| **Blinding of participants and personnel** | High risk. | Open label |
| *Detection bias* | | |
| **Blinding of outcome assessment** | High risk. | Open label |
| *Attrition bias* | | |
| **Incomplete outcome data addressed** | Low risk. | There is only one intervention including all participants |
| *Reporting bias* | | |
| **Selective reporting** | Low risk. | All prespecified outcomes were reported |
| *Other bias* | | |
| **Other source of bias** | High risk. | Quote : « [subjects] were free to move around within the Clinical Research Center during the study and had access to a stationary bicycle ergometer; however, physical activity was not directly monitored» |

| **Study : Levine et al. (2008)** | | |
| --- | --- | --- |
| **Entry** | **Authors’ judgement** | **Support of judgement** |
| *Selection bias* | | |
| **Random sequence generation** | Unclear risk. | No information |
| **Allocation concealment** | Unclear risk. | No information |
| *Performance bias* | | |
| **Blinding of participants and personnel** | High risk. | Open label |
| *Detection bias* | | |
| **Blinding of outcome assessment** | High risk. | Open label |
| *Attrition bias* | | |
| **Incomplete outcome data addressed** | Low risk. | Quote : « Ten subjects (5 women and 5 men) were lean (BMI<25 kg/m^2^) and 12 subjects (7 women and 5 men) were obese (BMI> 29 kg/m^2^) »  Numbers randomized into each intervention group are clearly reported |
| *Reporting bias* | | |
| **Selective reporting** | Low risk. | All prespecified outcomes were reported |
| *Other bias* | | |
| **Other source of bias** | High risk. | Quote : « subjects were instructed not to adopt new exercise practives and to continue their usual daily activities and occupation» |

| **Study : Levine et al. (1999)** | | |
| --- | --- | --- |
| **Entry** | **Authors’ judgement** | **Support of judgement** |
| *Selection bias* | | |
| **Random sequence generation** | Unclear risk. | No information |
| **Allocation concealment** | Unclear risk. | No information |
| *Performance bias* | | |
| **Blinding of participants and personnel** | High risk. | Open label |
| *Detection bias* | | |
| **Blinding of outcome assessment** | High risk. | Open label |
| *Attrition bias* | | |
| **Incomplete outcome data addressed** | Low risk. | Quote : « Sixteen nonobese adults (12 males and 4 females, ranging in age from 25 to 36 years) underwent measures of body composition and energy expenditure »  Numbers randomized into each intervention group are clearly reported |
| *Reporting bias* | | |
| **Selective reporting** | Low risk. | All prespecified outcomes were reported |
| *Other bias* | | |
| **Other source of bias** | Low risk. | Quote : « Altough we appreciated that volitional exercise might change in response to overeating, we viewed this as a behavioral rather then a physiological adaptation and so elected to eliminate it as a confounding » variable» |

| **Study : Muller et al. 2015** | | |
| --- | --- | --- |
| **Entry** | **Authors’ judgement** | **Support of judgement** |
| *Selection bias* | | |
| **Random sequence generation** | Unclear risk. | No information |
| **Allocation concealment** | Unclear risk. | No information |
| *Performance bias* | | |
| **Blinding of participants and personnel** | High risk. | Open label |
| *Detection bias* | | |
| **Blinding of outcome assessment** | High risk. | Open label |
| *Attrition bias* | | |
| **Incomplete outcome data addressed** | Low risk. | Quote : « Study 1 followed the original 6-wk intervention protocol »  Numbers randomized into each intervention group are clearly reported |
| *Reporting bias* | | |
| **Selective reporting** | Low risk. | All prespecified outcomes were reported |
| *Other bias* | | |
| **Other source of bias** | Low risk. | Quote : « A physical activity level of 1.4 was taken to resemble a sedentary lifestyle » |

| **Study : Pasquet et al. 1992** | | |
| --- | --- | --- |
| **Entry** | **Authors’ judgement** | **Support of judgement** |
| *Selection bias* | | |
| **Random sequence generation** | High risk. | Quote : « Nine lean young adult men volunteered for study throughout the Guru Walla session, having been fully informed of our goals and procedures » |
| **Allocation concealment** | Unclear risk. | No information |
| *Performance bias* | | |
| **Blinding of participants and personnel** | High risk. | Open label |
| *Detection bias* | | |
| **Blinding of outcome assessment** | High risk. | Open label |
| *Attrition bias* | | |
| **Incomplete outcome data addressed** | High risk. | Participant included in the analysis are not exactly those who were randomized into the trial. |
| *Reporting bias* | | |
| **Selective reporting** | Low risk. | All prespecified outcomes were reported. |
| *Other bias* | | |
| **Other source of bias** | Unclear risk. | No information about physical activity indication. |
|  | | |
| **Study : Siervo et al. (2008)** | | |
| **Entry** | **Authors’ judgement** | **Support of judgement** |
| *Selection bias* | | |
| **Random sequence generation** | Unclear risk. | No information |
| **Allocation concealment** | Unclear risk. | No information |
| *Performance bias* | | |
| **Blinding of participants and personnel** | High risk. | Open label |
| *Detection bias* | | |
| **Blinding of outcome assessment** | High risk. | Open label |
| *Attrition bias* | | |
| **Incomplete outcome data addressed** | Low risk. | Quote : « No subjects were excluded from the study for intercurrent adverse events. One subject did not complete the final OF period and ad libitum phase. » |
| *Reporting bias* | | |
| **Selective reporting** | Low risk. | All prespecified outcomes were reported. |
| *Other bias* | | |
| **Other source of bias** | Low risk. | Quote : « The volunteers were instructed to maintain their usual level of physical activity, and, except for the exercise performed in the metabolic chamber, deliberate additional exercise was not allowed ». |

| **Study : Ravussin et al. (1985)** | | |
| --- | --- | --- |
| **Entry** | **Authors’ judgement** | **Support of judgement** |
| *Selection bias* | | |
| **Random sequence generation** | Unclear risk. | No information |
| **Allocation concealment** | Unclear risk. | No information |
| *Performance bias* | | |
| **Blinding of participants and personnel** | High risk. | Open label |
| *Detection bias* | | |
| **Blinding of outcome assessment** | High risk. | Open label |
| *Attrition bias* | | |
| **Incomplete outcome data addressed** | Low risk. | Quote : « Sixteen nonobese adults (12 males and 4 females, ranging in age from 25 to 36 years) underwent measures of body composition and energy expenditure »  Numbers randomized into each intervention group are clearly reported |
| *Reporting bias* | | |
| **Selective reporting** | High risk. | Quote : «This preliminary study was used, not onlu to determine each individual’s « free-ranging » energy requirements, calculated as Ee within the chamber plus an estimated 25% for physical activity while outside the chamber (unpublished observation »  Data not available. |
| *Other bias* | | |
| **Other source of bias** | Low risk. | Quote :   1. « The five subjects were known to have maintained their body weight essentialy constant over 2 yr before the study» 2. « no vigorous physical activity was permitted in the chamber, spontaneous physical activity was estimated by radar » |

| **Study : Roberts et al. (1990)** | | |
| --- | --- | --- |
| **Entry** | **Authors’ judgement** | **Support of judgement** |
| *Selection bias* | | |
| **Random sequence generation** | Unclear risk. | No information |
| **Allocation concealment** | Unclear risk. | No information |
| *Performance bias* | | |
| **Blinding of participants and personnel** | High risk. | Open label |
| *Detection bias* | | |
| **Blinding of outcome assessment** | High risk. | Open label |
| *Attrition bias* | | |
| **Incomplete outcome data addressed** | Low risk. | Quote : «The subjects were 7 young and 9 older men of typical body weight and fat content»  Numbers randomized into each intervention group are clearly reported |
| *Reporting bias* | | |
| **Selective reporting** | Low risk. | All prespecified outcomes were reported |
| *Other bias* | | |
| **Other source of bias** | High risk. | Quote : « At the time of the study, all were either full-time students, individuals employed in sedentary occupations, or retired». |

| **Study : Weyer et al. (2001)** | | |
| --- | --- | --- |
| **Entry** | **Authors’ judgement** | **Support of judgement** |
| *Selection bias* | | |
| **Random sequence generation** | Unclear risk. | No information |
| **Allocation concealment** | Unclear risk. | No information |
| *Performance bias* | | |
| **Blinding of participants and personnel** | High risk. | Open label |
| *Detection bias* | | |
| **Blinding of outcome assessment** | High risk. | Open label |
| *Attrition bias* | | |
| **Incomplete outcome data addressed** | Low risk. | Quote : «Fourteen male subjects, seven Caucasians and seven Pima Indians participated in this study»  Numbers randomized into each intervention group are clearly reported. |
| *Reporting bias* | | |
| **Selective reporting** | Low risk. | All prespecified outcomes were reported. |
| *Other bias* | | |
| **Other source of bias** | Low risk. | Quote : « All subjects were abstained from strenuous activity » |
